# Mesenchymal stem cell-based therapy for non-healing wounds due to chronic limb-threatening ischemia: A review of preclinical and clinical studies

**DOI:** 10.3389/fcvm.2023.1113982

**Published:** 2023-02-01

**Authors:** Carlos Theodore Huerta, Francesca A. Voza, Yulexi Y. Ortiz, Zhao-Jun Liu, Omaida C. Velazquez

**Affiliations:** ^1^DeWitt Daughtry Family Department of Surgery, University of Miami Miller School of Medicine, Miami, FL, United States; ^2^Vascular Biology Institute, University of Miami Miller School of Medicine, Miami, FL, United States

**Keywords:** stem cell therapy, cell-based therapy, wound healing, diabetic wound, mesenchymal stem (stromal) cell, critical limb ischemia, chronic limb-threatening ischemia, Peripheral Arterial Disease

## Abstract

Progressive peripheral arterial disease (PAD) can result in chronic limb-threatening ischemia (CLTI) characterized by clinical complications including rest pain, gangrene and tissue loss. These complications can propagate even more precipitously in the setting of common concomitant diseases in patients with CLTI such as diabetes mellitus (DM). CLTI ulcers are cutaneous, non-healing wounds that persist due to the reduced perfusion and dysfunctional neovascularization associated with severe PAD. Existing therapies for CLTI are primarily limited to anatomic revascularization and medical management of contributing factors such as atherosclerosis and glycemic control. However, many patients fail these treatment strategies and are considered “no-option,” thereby requiring extremity amputation, particularly if non-healing wounds become infected or fulminant gangrene develops. Given the high economic burden imposed on patients, decreased quality of life, and poor survival of no-option CLTI patients, regenerative therapies aimed at neovascularization to improve wound healing and limb salvage hold significant promise. Cell-based therapy, specifically utilizing mesenchymal stem/stromal cells (MSCs), is one such regenerative strategy to stimulate therapeutic angiogenesis and tissue regeneration. Although previous reviews have focused primarily on revascularization outcomes after MSC treatments of CLTI with less attention given to their effects on wound healing, here we review advances in pre-clinical and clinical studies related to specific effects of MSC-based therapeutics upon ischemic non-healing wounds associated with CLTI.

## 1. Introduction

Peripheral arterial disease (PAD) is a major source of cardiovascular morbidity and mortality affecting more than 230 million people globally (1). For those with PAD, 11% develop its most severe form, chronic limb-threatening ischemia (CLTI), that confers 50% 5 year and 70% ten year mortality rates (2, 3). CLTI manifestations include rest pain and tissue loss from impaired wound healing due to reduced reperfusion and dysfunctional neovascularization (4). Patients with comorbid diabetes mellitus (DM) are up to 40 times more likely to develop CLTI (5, 6). Moreover, this population has a propensity for multifocal disease and aberrant immune function that can further impair wound healing compared to their non-diabetic counterparts (5, 6). Even when accounting for other baseline medical comorbidities and patient characteristics, patients presenting with ulcers in the setting of CLTI have a significantly increased odds of major extremity amputation [Odds Ratio (OR) 1.41] and mortality (OR 1.55) compared to patients with rest pain only (7). Treatment for CLTI consists mainly of risk factor mitigation with control of cholesterol and glycemic levels, smoking cessation, and endovascular or open surgical revascularization if feasible. Unfortunately, nearly 20–40% of patients may not respond to or have failed standard medical therapy or are unsuitable for revascularization (8–10). Given the high economic burden imposed on patients, decreased quality of life, and poor survival of those with CLTI, regenerative therapies aimed at the promotion of neovascularization to improve wound healing and limb salvage hold significant promise.

Under normal conditions, wound healing involves multifactorial, orchestrated self-repair mechanisms to restore the integrity of the mucosal and skin barriers. These processes become highly dysregulated in the hypoxic/ischemic milieu of CLTI extremity tissue and result in sustained wound disclosure and ulceration (11). Moreover, numerous studies have previously reported the refractory nature of ischemic wounds associated with CLTI to treatment, and the most common local wound care regimens appear to have only a minimal impact upon the size of the overall ulcer (11–14). Patients with persistent disease despite optimal medical therapy and surgical revascularization or who have contraindications to surgical intervention and are deemed “no-option” face major rates of amputation (46%) and mortality (50%) at 1 year, even worse than some malignancies (8–10). Consequently, there is a critical unmet need for newer vascular regenerative therapies. While previous reviews of the literature have focused on generalized applications and outcomes of MSC therapy for CLTI revascularization, little attention has been given to wound healing outcomes and tissue loss of CLTI as well as to integrate discussions of both preclinical and clinical advancements in this arena.

## 2. Properties and limitations of cell-based therapy

**Figure 1:**
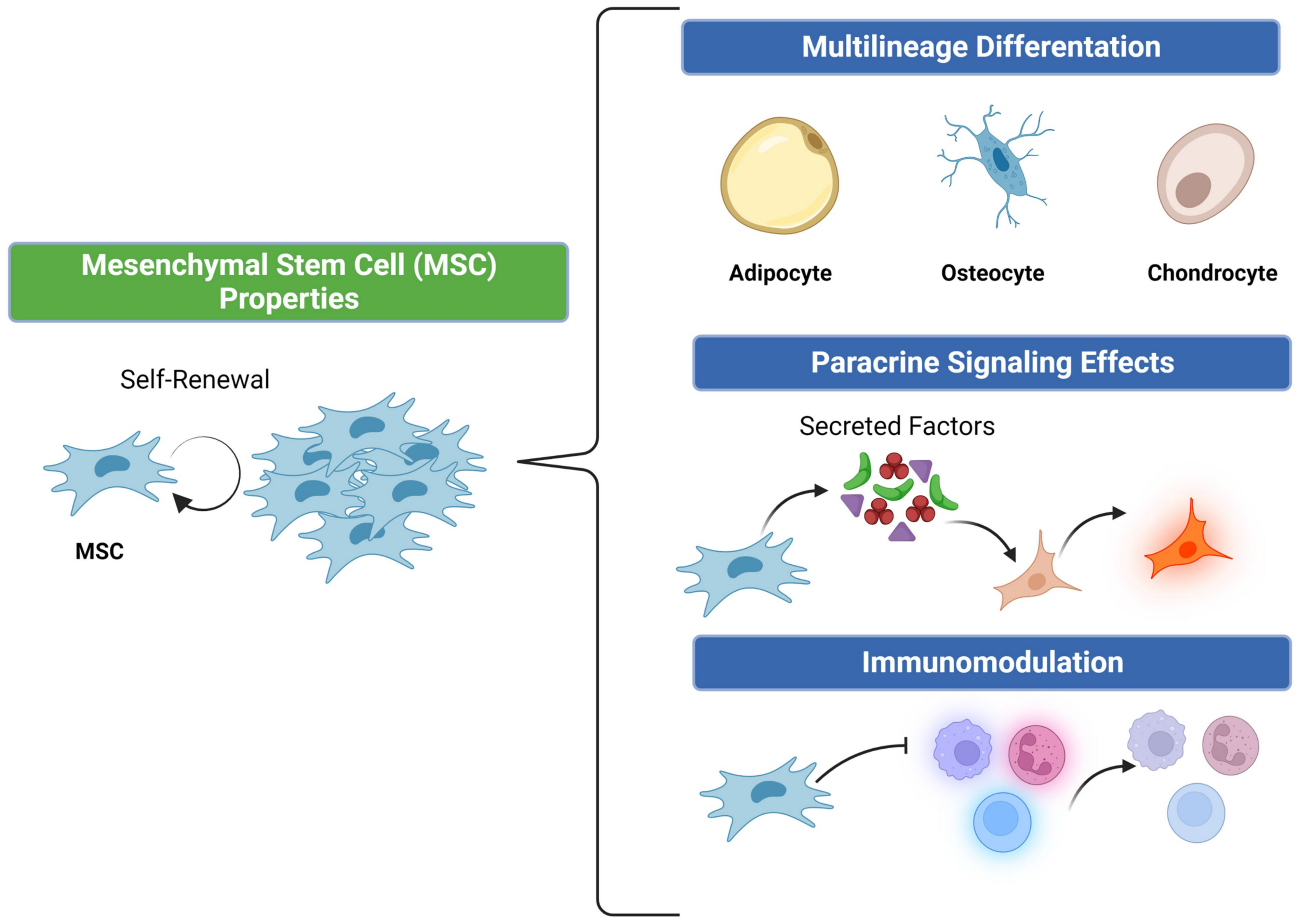
Properties of mesenchymal stem cells.

Cell therapy, specifically using mesenchymal stem/stromal cells (MSCs), is one such regenerative strategy to stimulate therapeutic angiogenesis. MSCs constitute an essential role in wound healing and angiogenesis due to their inherent potential for multilineage cell differentiation, ability to secrete soluble factors and exosomes (paracrine functions), and immunomodulatory and antibacterial activity (Figure 1) (15). These functions aid in the endogenous tissue repair milieu by recruiting cells involved in tissue repair and immunomodulation as well as through replacing resident cells in the wound environment (16). MSCs ability for self-renewal can allow for improved proliferation and replenishment of MSCs at target sites versus other cell lineages (16). Their low immunogenicity as well as immunosuppressive abilities help dampen the host immune response upon transplantation, which also makes MSCs an attractive therapeutic option compared to other cell types (17, 18). MSCs can be further harvested from a variety of tissue types including adipose, bone marrow, and placental/umbilical cord, which can also impact their intrinsic cell properties (15).

**Figure 2:**
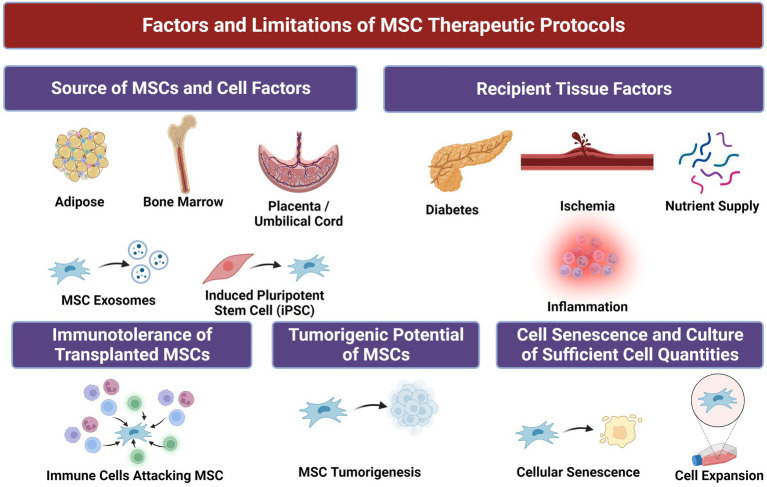
Factors and associated limitations of mesenchymal stem/stromal cells (MSCs) for therapeutic purposes.

**Figure 3:**
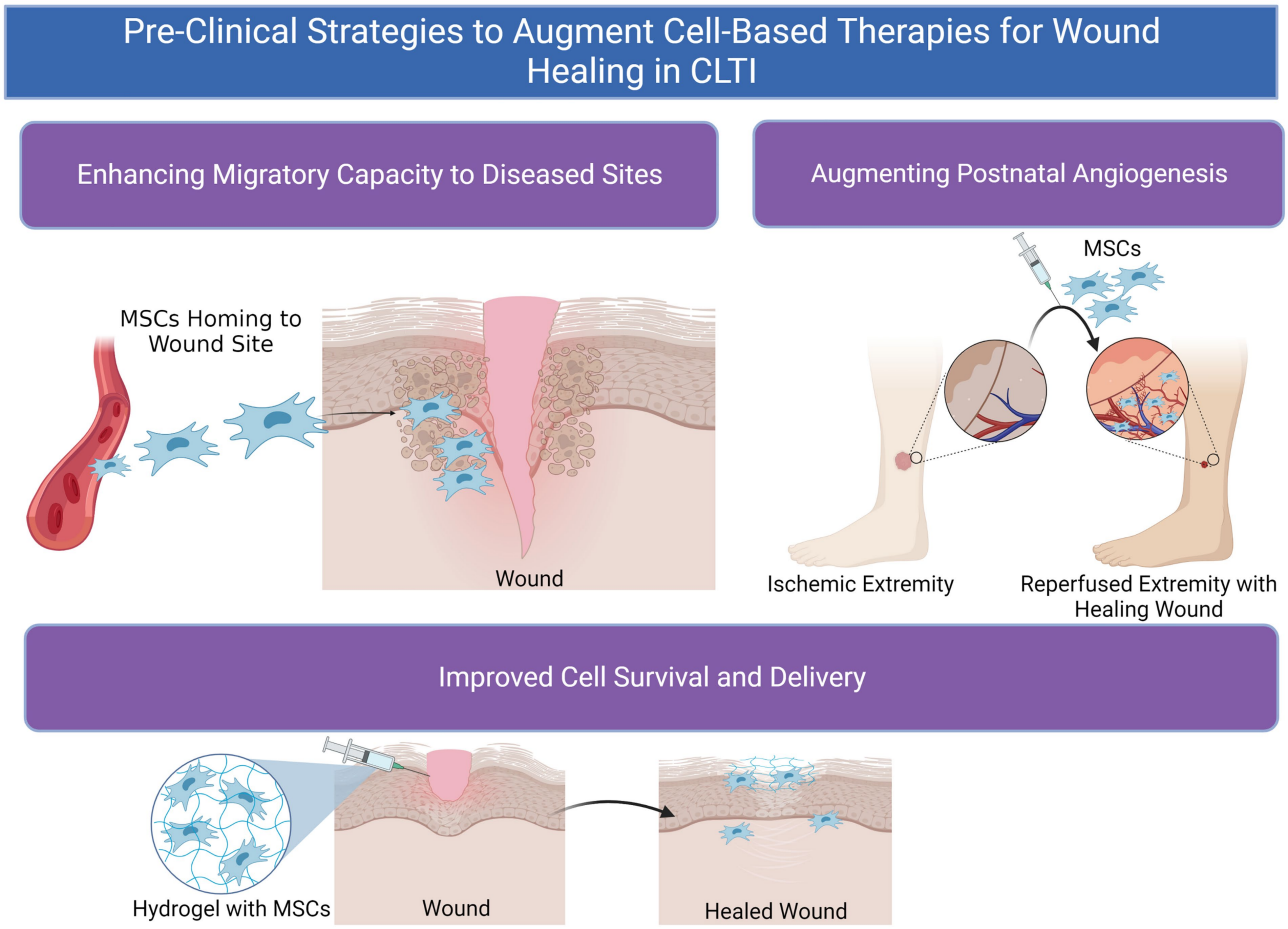
Pre-clinical methods to augment cell-based therapeutics for wound healing in chronic limb-threatening ischemia (CLTI).

However, unmodified MSCs have demonstrated only minor benefits with regards to amputation-free survival (AFS) rates in many clinical trials compared to placebo, which some posit may be due to mild potency of MSCs given (17, 19, 20). Syngeneic MSC populations harvested from donors with CLTI and/or concomitant diseases such as DM can suffer from reduced proliferative capacity and therapeutic potential compared to cells from healthy donors (21). Furthermore, the effectiveness of stem cell therapy is contingent upon effective engraftment and homing of MSCs to diseased tissues in order to restore function and homeostasis. Even when locally dispensed to sites of disease, MSC therapy is significantly limited by cell viability, retention, and decreased or nonspecific cell migratory capacity (22). Heterogeneity in donor MSC populations can also persist and result in issues with immunocompatibility between recipient and donor (23). These challenges are increasingly enhanced in diseased states, which can be inhospitable to MSCs due to tissue hypoxia, hyperglycemia, diminished blood flow, and wide-spread local inflammation in many disease states (Figure 2) (22, 24). Therefore, methods to optimize MSCs’ native functionality and delivery conditions with due consideration of the recipient tissue microenvironment to which they are implanted are needed to achieve maximum clinical efficacy (Figure 3).

## 3. Preclinical studies

### 3.1. Augmenting postnatal angiogenesis with MSCs

Tissue regeneration and neovascularization are intimately associated processes required for effective wound healing. Unfortunately, patients with CLTI and/or DM may have diseased MSC cell populations with reduced proliferative and angiogenic potential. As a result, strategies to enhance the pro-repair phenotype of MSCs in both healthy and diseased cells, such as through genetic engineering of cells to overexpress pro-angiogenic factors, can be utilized to improve vascular regeneration and wound healing in patients with CLTI (21). For example, Park et al. modified MSCs to overexpress Vascular Endothelial Growth Factor (VEGF) and subsequently utilized this MSC therapy in mice with surgically created hindlimb ischemia (25). This resulted in significantly improved revascularization and attenuated fibrosis and necrosis in ischemic limbs compared to treatment with untransfected cells, which may in part be due to heightened canonical VEGF/VEGFR signaling induction in neighboring endothelial cells although the authors did not explicitly examine cellular targets (25).

Quiroz et al. programmed murine bone marrow-derived MSCs (BM-MSCs) to overexpress an adhesion molecule, E-selectin, using an adeno-associated viral vector (24). Expression of E-selectin in MSCs resulted in the upregulation of nine proangiogenic genes *in vitro,* such as *Cxcl2,* involved in neovascularization as well as cellular trafficking and engraftment (24). In mice with surgically induced limb ischemia, treatment with E-selectin supercharged MSCs resulted in reduced muscle atrophy and augmented neovascularization compared to MSCs transduced with a control vector (24). The upregulation of these angiogenic gene profiles as well as the immunomodulatory effects of E-selectin modified MSCs in downregulating factors such as Tumor Necrosis Factor (TNF) in ischemic tissue may explain their effects on muscle fiber integrity and revascularization. Others have utilized priming methods to activate MSCs with soluble factors and induce cells towards a proangiogenic phenotype. For example, one study elucidated that MSCs cultivated in the presence of erythropoietin (EPO), a proangiogenic cytokine, rescued their ability to secrete VEGF and other paracrine factors in hyperglycemic environments (26). Those MSCs in hyperglycemic environments treated with EPO also demonstrated reduced secretion of TNFα *in vitro*, which was also thought to contribute to their improved anti-inflammatory and wound healing effects (26). Separate from CLTI, applications of these MSCs *in vivo* augmented wound healing of mice with diabetic foot ulcers (DFU) and improved their ability to differentiate into endothelial cells (26). Another study by Castilla et al. utilized stromal-derived factor-1α (SDF-1α) to activate MSCs from diabetic murine subjects, which resulted in augmented neovascularization in cutaneous wounds and faster healing in part due to SDF-1α-mediated upregulation of the Plasminogen/Plasmin signaling cascade (27).

Extending this line of reasoning to encompass combinations of multiple angiogenic targets may also be an elegant strategy to improve postnatal angiogenesis and subsequent wound healing. In one investigation, Jeong et al. manipulated MSCs using transcription activator-like effector nucleases (TALEN) genetic programming to concomitantly overexpress two factors: SDF-1α and chemokine granulocyte chemotactic protein-2 (GCP-2) (28). These modified MSCs demonstrated regenerative and angiogenic properties through their effects on both improved fibroblast migration and endothelial cell tube formation by *in vitro* scratch assay and Matrigel tube formation assays. Treatment with gene-edited MSCs augmented blood perfusion and prevented limb loss in a murine model of hindlimb ischemia compared to unmodified MSCs due to the enhanced proangiogenic milieu induced by GCP-2 and SDF-1α-mediated upregulation of factors such as Angiopoietin 1 (Ang1) and VEGF (28). Kerstan et al. utilized hypoxic incubation of skin-derived MSCs to induce upregulation of the transcription factor hypoxia-inducible transcription factor 1α (HIF-1α) (29). This further engaged canonical VEGF and angiogenic signaling pathways among MSCs to enhance capillary proliferation and vascularization in the ischemic muscle tissue of mice with hindlimb ischemia (29). Overall, continued preclinical studies to target postnatal angiogenesis with MSC therapy may provide future hope for clinical therapeutics to augment healing for patients with tissue gangrene and ischemic wounds. However, activation/priming MSCs by soluble factor(s) *in vitro*/*ex vivo* can also raise concerns about increased inflammation and potential tumorigenesis, which should be considered to optimize future therapeutic strategies (30).

### 3.2. Enhancing migratory capacity of MSCs to disease sites

Another major limitation to the effectiveness of MSCs to repair wounded tissue can be their innate ability to home and survive at sites of disease. Recruitment of circulating stem and progenitor cells such as MSCs to wounded tissue relies on critical interactions between cell populations and extracellular matrix factors (ECM) such as hyaluronan (HA), which are substantially upregulated in wound beds (31). These interactions can be exploited to enhance the migratory potential of MSCs through priming with cytokines. Zhu et al. demonstrated that culture of MSCs with platelet-derived growth factor (PDGF) led to overexpression of cell surface CD44, a factor mediating migration *via* HA-binding (32). This relationship could be negated by CD44 inhibitory antibodies, and the upregulation of CD44 on MSCs led to substantially improved migratory capacity of MSCs (32). Other investigators have shown that the defective phenotype of adipose-derived (AD-MSCs) from diabetic mice is in part due to impaired PDGF signaling and subsequent ERK 1/2 pathway activation, which results in decreased proliferative and migratory capability. Utilizing a cutaneous wound healing model, these authors elucidated that priming of adipose-derived MSCs (AD-MSCs) from diabetic mice with PDGF rescued their migratory potential and multilineage differentiation capacity *via* PDGF associated signaling pathways (33).

Priming MSCs can further enhance their ability to recruit other cells to wound sites and thereby impact tissue regeneration. Compared to unmodified MSCs, Castilla et al. found that co-culture of BM-MSCs derived from diabetic mice with SDF-1α led to accelerated wound closure and endothelial progenitor cell recruitment in a mouse model of diabetic wound healing *via* downstream upregulation of Ephrin Receptor B4 and plasminogen (27). Using a viral vector to induce C-X-C Motif Chemokine Receptor (CXCR) 6 receptor expression in BM-MSCs, Dhoke et al. investigated the interaction between CXCR6 and its cognate ligand CXCL16 as a potential mechanism to enhance MSC engraftment to wound sites (34). Potentiation of the CXCL16-CXCR6 axis resulted in induction of cell migratory pathways *via* activation of factors including Src, focal adhesion kinase (FAK), and extracellular signal-regulated kinases 1/2 (ERK1/2). In turn, engineering MSCs with CXCR6 overexpression augmented their recruitment and engraftment in wounds and subsequently promoted re-epithelialization and neovascularization in mice with diabetic wounds. Further areas of preclinical research targeting the migratory potential of MSCs may provide future hope for patients with ischemic wounds in which circulation and exposure of diseased tissue to cell populations is significantly impaired.

### 3.3. Improving MSC survival and delivery

Another limiting factor to the efficacy of cell therapy is the host environment to which cells are transplanted, which can be significantly inhospitable in diseased environments. By utilizing protective cell delivery systems and vehicles such as hydrogels, the survival and retention of MSC therapy can be augmented. For example, Wang et al. designed a methyl-cellulose-based hydrogel to deliver placental-derived MSCs (PD-MSCs) at hypothermic temperatures around 32°C (35). This is particularly advantageous given that the temperature of ischemic wounds and tissue in patients with CLTI is known to be significantly lower compared to well-perfused tissue (35). As a result, PD-MSCs delivered in this hydrogel improved limb revascularization, cell viability and retention, and attenuated muscular atrophy and death in a hindlimb ischemia model, which may be due to the increased secretion of VEGF and downregulation of genes related to apoptosis (*BAX* and *Bcl-2*) among MSCs in hydrogel (35). Others have further exploited the design of hydrogel systems to be more protective of the transplanted cells delivered such as through the development of small-molecule hydrogels (36). Huan et al. have shown that small-molecule hydrogels associated with disulfide bonds improved engraftment of MSCs to diseased tissues due to the increased mechanical stiffness and protection from shear stress during injection associated with this design (36). Amplified engraftment of PD-MSCs in their study led to heightened reperfusion and muscle tissue regeneration in mice with hindlimb ischemia (36). Other autologous substrates such as platelet-rich plasma (PRP) hold significant promise for tissue regeneration given their numerous bioactive molecules and signaling factors associated with platelet alpha granules (PDGF, VEGF, etc.) (37). Co-administration of PRP with PD-MSCs in one study improved implantation of PD-MSCs, which resulted in accelerated wound closure and tissue regeneration in patients with DFUs (38). These authors further utilized a gelatin nanofibrous scaffold for the delivery of MSCs, which enhanced proliferation of MSCs *in vitro* and may account for more subsequently incorporated MSCs at wound sites (38). This was not studied in patients with CLTI but could be considered in future work for patients with CLTI and comorbid DM who have wounds.

A novel and exciting field within cell-based therapy is the concept of targeted cell delivery, which can enhance therapeutic efficacy and off-set some downsides of systemic cell therapy. Investigators have begun utilizing adhesion molecules to direct MSC migration and specify homing to wound areas (22, 39, 40). For instance, E-selectin, an inducible cell-adhesion molecule with pro-angiogenic signaling effects, along with its ligands, including CD44, CD62 and E-selectin ligand (ESL), have been shown to be upregulated on vasculature in ischemic tissue (41, 42). Liu et al. demonstrated that systemic administration of pre-modified MSCs on which cell surfaces are coated with E-selectin-conjugated dendrimers directed MSC homing to wound sites mediated by enhanced interaction of E-selectin/ligands, resulting in increased tissue repair in an ischemic diabetic wound healing model (22). This physical cell modification led to efficient trafficking of MSCs to wound areas without indiscriminate distribution to other organs even when administered systemically (22). Hematopoietic stem cells have previously been known to demonstrate a specific glycoform of CD44 referred to as hematopoietic cell E−/L-selectin ligand (HCELL) that function as a highly potent E-selectin ligand to improve their recruitment within bone marrow compartments (39). Sackstein et al. modified surface CD44 on MSCs into HCELL and conferred significantly improved tropism of these cells to bone, which can provide a method or “roadmap” for targeted cell delivery to areas with heightened ESL expression such as in ischemic tissue (40, 43).

Another emerging area of MSC-related therapies for vascular regeneration and wound healing is the development MSC spheroids. Three-dimensional culture systems can be utilized to generate clusters of MSC cells in spherical aggregates, which more closely recapitulates the native MSC niche and improves the retention and expansion of MSCs for therapeutic applications (44). Forced aggregation of cells in spheres or clusters can potentiate their individual cell capacity through differential cell polarization, adhesion molecule expression, and physical interactions between cells (44). Yang et al. demonstrated that delivery of MSC spheroids *via* a chitosan scaffold substantially augmented the proliferation and expression of paracrine factors from MSC → s such as VEGF, Ang1, CXCR4, and SDF-1α (45). This resulted in accelerated wound closure and enhanced vascular density in wounds delivered to diabetic mice compared to standard MSCs cultured in two dimensional monolayers (45). Another emerging advantage of spheroid culture systems is the ability to create aggregates containing MSCs along with other beneficial cell types. Bayaraa et al. recently reported the creation of a multicellular delivery system containing both MSCs and endothelial cells (46). This cell system resulted in improved cell proliferation and increased secretion of angiogenic factors (VEGF1, HIF1α) *via* APE/Ref-1 signaling. When delivered to the ischemic hindlimb of mice, treatment with this MSC/endothelial cell platform resulted in enhanced blood reperfusion and limb salvage thought to be due to the improved proliferative and angiogenic profile of these cells (46). Future studies such as these may continue to elucidate the optimal delivery method or combination of cell culture and administration methods to optimize MSC therapy for patients with ischemia and wounds associated with CLTI who often have concomitant DM.

## 4. Clinical studies

The promising results obtained from pre-clinical investigations on progenitor cells as potential therapeutic tools for wound healing and revascularization in patients with CLTI have led to several clinical studies over the past two decades. While the goal of therapy is to augment limb salvage rates, CLTI patients quality of life is severely impacted in large part by rest pain, non-healing ulcers, and walking disability due to claudication. Subsequent endpoints utilized by clinical trials to evaluate the efficacy of treatment include wound area closure and/or size reduction, and change in pain and Quality of Life (QoL) scores (47). Hemodynamic metrics such as ankle-brachial index (ABI) or transcutaneous oxygen pressure (TcPO2) are also frequently monitored during clinical studies as surrogates of enhanced limb perfusion in addition to angiographic evidence of new blood vessel development (48). The first stem cell trials for CLTI pioneered by Tateishi-Yuyama et al. reported the safety and efficacy of bone marrow-derived mononuclear cells (BM-MNCs) in patients (TACT trial) (49). However, limitations and risks related to the use of MNCs were reported by different institutions primarily due to the broad spectrum of cells contained in the bone marrow with a small percentage of therapeutically active cells which have the potential to differentiate into undesirable cells and tissue types (20, 50).

The renewed focus on stem cells’ multipotency and paracrine secretion of angiogenic factors gave rise to clinical trials in CLTI patients *circa* 2008 using modified MSCs (51). Lu et al. in a randomized control study of patients with DM and severe CLTI (defined as ABI < 0.6), demonstrated the safety profile of autologous BM-MSC therapy and significant augmentation of ulcer healing rates in this patient population (51). In their subsequent comparative study between BM-MSCs and BM-MNCs, they also reported higher efficacy of MSCs with a better ease of use and reduced risk for patients given that BM-MSC cytotherapy required a lower aspiration volume of bone marrow compared to BM-MNCs. This resulted in decreased cumulative anesthetic exposure for a patient population already at increased risk of perioperative cardiopulmonary complications, as well as improved tolerance of the procedures by patients (52). As a result, it is necessary to understand how the differential selection and administration routes of MSCs can demonstrably affect clinical outcomes both in terms of neoangiogenesis and wound healing in patients with CLTI and comorbidities such as DM.

### 4.1. Marker-selected cell types and tissue sources

Functional and phenotypic characterization of MSCs from a rich reservoir such as bone marrow necessitates an underlying knowledge of the mechanisms employed for molecular education of progenitor and stem cells (51). A less heterogenous pool of cells could potentially increase the proportion of “active” cells responsible for the beneficial effects leading to neovascularization and wound healing (42). MSCs were first distinguished from hematopoietic stem cells by the lack of CD34 cell surface marker and increased expression of CD90 and CD105 (50, 53, 54). Dash et al. in a randomized clinical trial on CLTI in patients also suffering from concomitant DFUs or Buerger’s disease, demonstrated significant reduction in ulcer size compared to those in the control group using autologous BM-MSCs characterized by cell-surface markers for CD90^+^, CD105^+^, and CD34^−^ expression (55). However, this study was non-blinded.

In the double-blind randomized multicenter phase II RESTORE CLTI trial study, Powell et al. further demonstrated that it was safe to transplant no-option CLTI patients with expanded CD90^+^/CD14^+^ MSCs cells using the Ixmyelocel-T cell population (56). Selecting for these markers allowed for a smaller volume collection of bone marrow as well as a more standardized and potent therapy than fresh bone marrow preparations (56). Gupta et al. in a phase I/II randomized, double-blind placebo controlled multi-center trial of IM injection in CLTI patients comparing allogeneic CD73^+^/CD90^+^ BM-MSCs and CD34^+^/CD45^+^ BM-HSC (Stempeucel), reported no difference in adverse events (AEs) between placebo and Stempeucel treated groups (57). Results of the subsequently completed phase II and phase IV studies are not published yet. However, multiple discordant approaches used by clinical investigators to purify, characterize, and expand MSCs from the broader pool of stem cells continue to render the interpretation of clinical trial outcomes quite challenging (49, 51, 58). Consequently, the International Society for Cellular Therapy (ISCT) in 2016 defined the minimum criteria required to identify human MSCs: (1) cells must have the capacity to adhere to plastic, (2) must express CD73, CD90, and CD105 but lack expression of the hematopoietic lineage markers CD45, CD34, CD14 or CD11b, CD79α or CD19 and HLA-DR surface molecules, and (3) must be able to differentiate into adipocyte, chondrocyte, and osteoblast lineages *in vitro* (58).

The possibility of isolating allogeneic cells using these markers could also provide patients with healthy cells not affected by their native comorbidities such as DM. Furthermore, extraction of MSCs from tissue sources that do not require invasive procedures, such as bone marrow aspiration for BM-MSCs, may provide another attractive option to harvest allogeneic cells from donors. Clinical trials utilizing cells from adipose tissue, nervous tissue, amniotic fluid, umbilical cord, placenta, menstrual blood, and dental pulp are currently on-going (Table 1). AD-MSCs are an attractive tissue source for MSCs given the relatively high quantity of cells that can be harvested using minimally invasive techniques and their ability to rapidly expand in culture (53, 59, 60). The safety and effectiveness of AD-MSCs administered *via* IM transplantation in CLTI patients suffering from DM and Buerger’s disease was demonstrated by Lee et al. in a pilot study (60). Losordo et al. similarly provided evidence that IM administration of autologous CD34^+^ cells in those with moderate or high risk CLTI was safe to use in this patient population with improved wound healing and functional outcomes (61). In the ACellDREAM phase I clinical study, Bura et al. reported no AEs and augmented wound healing after autologous AD-MSCs were administered intramuscularly in diabetic and non-diabetic no-option CLTI patients (62). Although as a Phase I study it was non-blinded, these results are encouraging given that the number of AD-MSCs is greater than BM-MSCs in addition to the fact that adipose tissue is more readily available and in higher quantity than bone marrow (63). Pluristem Therapeutics is currently evaluating placenta expanded-PAD (PLX-PAD) cells in the treatment of CLTI in a double-blind, multinational, randomized, placebo-controlled fashion in their phase III trial (64, 65). Yang et al. reported in a small sample (*n* = 8) phase I clinical study that umbilical cord blood mesenchymal stem cells (UCB-MSCs) administered *via* intramuscular injection is a safe and well tolerated treatment for no-option patients with end-stage CLTI in addition to improvement of ulcer healing (66). It is important to note that cells used in these studies were not necessarily fulfilling the minimum criteria for MSCs according to the ISCT. Comparative studies between cell populations that do and do not meet the minimum criteria have yet to be done in order to establish a more standardized approach in clinical trials. Furthermore, study design with regards to placebo utilization and double-blinding intervention are critical to achieving reasonable conclusions as to the merits of MSC therapy for wound healing in CLTI patients.

**Table 1:** Clinical trials utilizing cell-based therapy for patients with chronic limb-threatening ischemia (CLTI).

Trial ID	Type of study	Patient enrollment (*N*)	Phase	Type of cells used	Cellular markers	Route	Outcomes	Wound healing outcome	Follow-up (months)	References
NCT00883870	Randomized, Double Blind, Multicentric, Placebo Controlled	CLI (20)	I/II/completed	Stempeucel^®^ (allogeneic BM-MSCs)	CD73, CD90, CD166, CD106, CD34, CD45, CD14, CD19, HLA-DR and CD133	IM	AEs, ABI, AR, NRS, UH	Photography by independent physicians	6	57
NCT01065337	Randomized	DFU (30)	II/completed	BM-MNCs and BM-MSCs	CD90+	IM	ASM, AFS, ABI, UH TcPO2,	Complete UH rate, no ipsilateral relapse	12	54
NCT01484574	Non-randomized, Open Label, Multicentric, Dose Ranging	CLI, Buerger’s disease (90)	II/completed	Stempeucel (allogeneic CD90+ BM-MSCs)	CD 90+	IM	UH, NRS, PWD, AFS, ASM, ABI, TcPO2, RA, NRS	UH or reduction of ulcer area in target limb	24	57
NCT01456819	Randomized, single blinded	CLI (50)	II/unknown	BM-MNCs or BM-MSCs	Heterogenous	IM	UH, VAS, DSA, ETT	Change in UH	12	Not provided
NCT01483898	Multicenter, Randomized, Double-Blind, Placebo-Controlled, Parallel Group Study	CLI (41)	III/completed	Ixmyelocel-T	CD90+ BM-MSC and CD45+/CD14+ activated macrophages	IM	AFS at 12 months	CWC by month 12	18	Not provided
NCT03042572	Double-blind, Randomized, Placebo-controlled	PAD, CVD (66)	II, III	(allogeneic) BM-MSCs	Heterogenous	IM	VAS, ABI, TBI, PWD, UH	Changes in number and extent of leg ulcers	6	69
NCT03455335	Uncontrolled, Non-randomized	CLI (12)	Ib/completed	Autologous BM-MSCs	Heterogenous	IM	AEs, AFS, AR, ABI, TcPO2, NRS, UH	Surface area from baseline as measured by ImageJ software / complete UH	12	84
NCT01257776	Prospective, multicenter, open, randomized, parallel-group controlled study	CLI, Diabetes (36)	I, II/completed	AD-MSCs or MNCs (CeTMAd)	Heterogenous	IA	Neovasculogenesis, AEs	UTCS at target limb	12	Not provided
NCT01745744	Prospective, Multicenter, Open, Randomized, parallel-group controlled	CLI (33)	I,II/completed	MSCs From Adipose Tissue (CeTMAd)	Heterogenous	IA	ABI, TcPO2, Score of Pain, AEs, AFS, AR, PS, QoL, ASM, TWD	UTCS	12	Not provided
NCT01302015	Single Group Assignment	Buerger’s Disease (15)	NA/completed	RNL-Vascostem^®^ (Ad-MSCs)	Heterogenous	IM	AEs, TWD, DSA, ABI, TcPO2, PWD	VAS	6	59
NCT02145897	Multicentric, Phase I/II, Open Label, Randomized	CLI (60)	I, II/unknown	Ad-MSCs	Heterogenous	IM/IV	AEs, ABI, TcPO2	Change in the number of debridements and wound coverage changes. Percent complete UH.	9	Not provided
NCT04661644	Non-Randomized	CLI (20)	I, IIa/recruiting	Ad-MSCs	Heterogenous	IM	PWD, ABI, TBI, MTD	VAS, Change in US	6	Not provided
NCT03267784	Interventional	Diabetic Neuropathic Ulcer (23)	I, IIa/completed	ABCB5^+^ MSCs	CD31^+^	Wound Surface	AEs, ABI, NRS, AR	Percent wound surface area reduction	12	29
NCT01216865	Randomized	CLI, DFU (50)	I, II/unknown	UCB-MSCs	Heterogenous	IM	Angiogenesis, ABI, UH, PWD, AR	US, Wound Stage	6	Not provided
NCT01859117	Non randomized	PAD, DFU (15)	I/completed	Cenplacel (PDA-002) (P-MSCs)	Heterogenous	IM	AEs, MTD, ABI, TBI	Not Stated	24	85
NCT03006770	Randomized, placebo-controlled, parallel group, multicenter,	CLI (213)	III/active, not recruiting	PLX-PAD (P-MSCs)	“PLX-PAD cells exhibit membrane marker expression typical of classical mesenchymal stromal cells”	IM	MA, DR, NRS, UH	Complete UH	36	65
NCT00951210	Non-Randomized	PAD,PVD, CLI (12)	I/completed	Placenta derived MSC (Biological: PLX-PAD)	Heterogenous	IM	AEs, AI, rehospitalization incidence	Not Stated	Not Stated	Not provided
NCT01386216	Non-Randomized	CLI, PVD (18)	I/completed	BM-MSCs	Heterogenous	IM	ABI, TcPO2, SPP, VAS, QoL	Not Stated	12	Not provided
NCT00616980	Randomized	PAD, PVD, CLI (28)	I, II/Completed	BM-MSCs	CD34+	IM	AEs, UH Change in Rest Pain, UH, AR	Not Stated	6	61
NCT01686139	Single Group Assignment	T1-DFU, T2-DFU (12)	I/completed	ABMD-MSC	Heterogenous	IM	AEs, UH	UTCS	Not Stated	50, 54, 55,
NCT03239535	Non-Randomized	CLI (40)	I,II/completed	Allogenic BM-MSCs	Heterogenous	IM	AEs, AFS, AR, TcPo2, ABI, PS, QoL, ASM, TWD	Assessment of the severity of trophic ulcers	12	Not provided

Adverse events (AEs), allogeneic bone marrow derived mesenchymal stromal cells (ABMD-MSC), ankle-brachial index (ABI), adipose-derived MSCs (AD-MSCs), amputation incidence (AI), amputation-free survival (AFS), amputation rate (AR), angiographic score of MRA (ASM), bone marrow-derived mononuclear cells (BM-MNCs), bone marrow-derived MSCs (BM-MSCs), complete wound closure (CWC), death rate (DR), diabetic foot ulcer (DFU), diabetes mellitus (DM), digital subtraction angiography (DSA), exercise treadmill test (ETT), maximum tolerated dose (MTD), mesenchymal stem cells (MSCs), numeric rating scale (NRS), pain free walking distance (PWD), pain scale (PS), peripheral arterial disease (PAD), placental-derived MSCs (PD-MSCs), quality of life (QoL), skin perfusion pressure (SPP), transcutaneous oxygen pressure (TcPO2), treadmill walking distance (TWD), umbilical cord blood mesenchymal stem cells (UCB-MSCs), ulcer healing (UH), University of Texas Diabetic Wound Classification Scale (UTCS), visual analog score (VAS).

### 4.2. Administration route

Another significant consideration for clinical studies of cell-based therapy is the route of cell administration. Direct introduction of cells into the injured tissue by intramuscular (IM) administration has been the main method for delivery of MSCs in most clinical trials. This route avoids risks that can be associated with more invasive endovascular administration such as injury to surrounding neurovascular and muscular structures, use of contrast reagent in patients that often suffer from compromised renal clearance (63), microthrombosis in diabetic patients (67), and potential tumorigenesis (50). In the RESTORE-CLTI trial, Powell et al. demonstrated that IM injection of Ixmyelocel-T including stem and progenitor cells is safe and well-tolerated with no significant difference in AEs compared to placebo (56). Concurrently, Walter et al. in the PROVASA trial investigated intra-arterial (IA) injection of progenitor cells in a randomized placebo-controlled study (68). This work elucidated dose-dependent effects of ulcer healing and improved rest pain compared with placebo without any difference in limb amputation rates compared to the placebo cohort. In a similar manner, Teraa et al. in the randomized, double-blind, placebo-controlled JUVENTAS trial utilizing repetitive transcutaneous intra-arterial treatments with BM-MNCs found no significant differences in ulcer healing or amputation rates between groups (69). While safety has been demonstrated *via* IM routes with significant improvement of wound healing (20), no significant difference in effectiveness was reported by Van Tongeren et al. or by Klepanec et al. in their comparative studies between IM and IA route of BM-MNCs (70, 71). Although study design (blinded versus non-blinded placebo-controlled trials) has been heterogeneous among these studies, they have shown a lack of adverse effects associated with IA and IM injection.

In a meta-analysis from Rigato et al., IM implantation appeared more effective than IA infusion with improved wound healing and amputation rates (72). In addition to the vascular regenerative and immunomodulatory characteristics of MSCs, their paracrine activity in the hostile tissue microenvironment of non-healing ulcers that lack a favorable vascular supply supports the use of IM versus IA administration (63). The multicenter, randomized, double-blind placebo-controlled phase II/II SAIL clinical trial will assess the safety and effectiveness of 30 IM injections of BM-MSCs in no-option CLTI patients (73). Kerstan et al. similarly demonstrated topical application of MSCs to the wound surface of patients with therapy resistant chronic lower extremity ulcers that resulted in improved wound closure and pain relief (74). Although DFUs constitute a separate clinical entity from CLTI, others have further demonstrated the safety and efficacy of UCB-MSCs applied topically to DFUs in achieving wound closure in a single phase I pilot study in no-option CLTI patients with concomitant DFUs (75). Overall, newer investigations of cell-based therapy in these patients should continue to explore methods to optimize the route of administration taking into full consideration the host tissue-level factors to augment MSC delivery and retention.

### 4.3. Investigations into CLTI patients with concomitant diabetic foot ulcers

A highly prevalent comorbidity in patients with CLTI is glucose intolerance and fulminant DM, and the use of MSCs from other sources than autologous progenitor/stem cells could be particularly relevant for DFU since MSC populations from diabetic patients exhibit reduced proliferative and angiogenic potential (22, 27). However, it is important to clarify that while CLTI can certainly hasten and concomitantly exist in the setting of DFUs, DFUs constitute a separate entity resulting from microvascular changes, neuropathy, and immunomodulatory effects due to hypergylcemia (51, 63). Effects of type 2 DM on different sources of autologous cells used in clinical therapy are well summarized in a diabetes-associated CLTI cell therapy review by Magenta et al. (63) These effects are thought to be mainly induced secondary to the increased inflammation and oxidative stress in diabetic wound beds due to environmental hyperglycemia (51, 63). DFU is a major complication of diabetes, and it is estimated that 84% of limb amputation in the diabetic population are preceded by a DFU (67). Lu et al. demonstrated from their investigations into DFU over the past decade that healing rates of ulcers in patients receiving BM-MSCs were significantly higher than placebo at 1 month after transplantation as well as compared to at 3 months compared to patients receiving BM-MNCs (76). Furthermore, the healing rate of ulcers reached 100% in the BM-MSC cohort earlier than patients receiving BM-MNCs (76).

The use of autologous MSCs in clinical studies on diabetic CLTI patients have shown significant improvement in rates of overall wound healing (60, 77). Lu et al. have shown that over a longer period limb amputation rates were lower in the BM-MSC group compared to the BM-MNC group, and the ulcer recurrence rate in the BM-MSC group was significantly lower than in the placebo group for 3–6 months after ulcer healing (64). These results are in concordance with Chen et al. who were able to show that compared to BM-MNCs, treatment of DFU with autologous BM-MSCs had superior outcomes than autologous BM-MNCs in the short and long term (55, 76, 77). These differences could be attributed to the paracrine and the vasculoregenerative features of MSCs, which are currently still in investigation in pre-clinical studies (63). Meanwhile, these results represent encouraging progress towards the treatment of CLTI patients with comorbid DM with either autologous or allogeneic MSCs isolated from different sources other than bone marrow. Other tissue sources could circumvent the invasive nature of bone marrow aspiration that requires anesthesia in a patient population predisposed to perioperative complications as well as allow the option of receiving cells from healthy donors. Current clinical trials are ongoing for diabetic wound healing therapies with MSCs from different sources. Investigations on MSCs from Adipose Tissue (CeTMAd) are underway in a phase I/II trial (NCT01257776, NCT01745744; Table 1). Kerstan et al. have recently completed a phase I/II randomized controlled trial, to test the safety of GMP-manufactured ABCB5^+^ dermal MSCs topical application to DFU (NCT03267784; Table 1) model after promising pre-clinical trials in the mouse model (29). Future studies should also examine similar MSC-based applications in CLTI patients with DM to ascertain whether similar beneficial effects for patients with DFU can be more broadly applied.

## 5. Conclusions and future perspectives

Those with tissue ischemia and loss secondary to CLTI constitute a vulnerable group of patients who face significant risks of amputation and mortality, which are further compounded in the presence of concomitant medical comorbidities such as DM (8–10). There continues to be an unmet need for clinical therapies and treatments to improve outcomes in patients who fail standard medical and surgical interventions. One rapidly evolving area with emerging potential for patients with this disease compared to traditional treatment regimens is MSC-based therapy. Pre-clinical studies have demonstrated targets to potentiate MSCs role in postnatal angiogenesis and tissue regeneration. Improving the migratory capability of MSCs to home to ischemic wound sites has shown promise to enhance their ability to participate in tissue regeneration. Local engraftment and survival of MSCs, especially in tissues with relative hypothermia and diminished blood supply, can be further improved through mechanical cell delivery systems and culture of aggregate 3D MSC spheroids. Combinations of these approaches may be elucidated in future studies to harness the maximum therapeutic effects of MSCs for clinical applications (78). Furthermore, future work should consider the ability of other cell-based therapies such as endothelial progenitor cells and mononuclear cells alone or in combination with MSCs for clinical revascularization strategies (47).

Ongoing clinical trials may offer hope to a patient population that continues to expand owing to the increasing prevalence of PAD, DM, and cardiovascular disease (2, 6). Even though amputation rates have not been significantly impacted compared to placebo, several important issues should be taken into consideration (11, 76, 79). Given the frailty of this patient population, studies have been of low power due to decreased sample sizes and subsequent interpretation of long term effects remains challenging (64). MSC treatments have demonstrated superior results compared to MNC treatments with partial improvements in wound healing and amputation rates (76, 77). The recent criteria for MSC characterization may help investigators standardize future studies leading to less heterogeneity between clinical trials and lead to the possible identification of active components at play in the process of wound healing in CLTI patients. Studies on the use of allogeneic cells from different sources more readily available than BM could offer a less invasive treatment to patients as well as treatment with healthier cells than the autologous cells of diabetic patients (67). Thus far, IM appears to be an optimal route of administration of the MSCs to the wound bed compared to IA administration in many studies. However, long term follow-up has shown that therapeutic effects of the cells can be transient (76). Dose and frequency of treatment have yet to be extensively tested (48, 63, 67). Dermal application is another method of application particularly for DFU that is currently under study by Kerstan et al. (NCT03267784; Table 1), and future studies should investigate the application of treatments used for DFUs in the population of CLTI patients with DM (29).

Future potential options for study include the potential of MSCs as an adjunctive therapy to standard revascularization options as well as combinations of systemic and topical cell therapy administration. Perhaps most importantly, few true double-blind placebo-controlled trials have been performed to examine MSC therapy, which is critically needed to fully appraise the clinical value of cell-based therapy for wound healing in CLTI patients. Scaffolding or 3D spheroids could possibly palliate the transient effect with a delivery method that could be sustained for longer periods of time (45, 47). The numerous proangiogenic cytokine and signaling factors in extracellular microvesicles make MSC-derived exosomes another potential strategy to induce limb reperfusion and neovascularization (80, 81). Moreover, studies on MSC paracrine functions using microRNA from cell exosomes have begun to show encouraging pre-clinical results (59, 63, 82). Emerging gene therapies to target angiogenic and tissue regeneration pathways within the recipient wound site through direct gene upregulation and/or silencing may also provide new alternatives and adjuncts to current cell therapies (83). Although significant progress has been made in the clinical arena, future methods and approaches to exploit the therapeutic nature of MSCs are ultimately needed to further optimize cell-based treatment and revascularization strategies for patients with CLTI (84–86).

## Author contributions

CH, FV, YO, Z-JL, and OV: study conception and design, analysis and interpretation of data, drafting of manuscript, and critical revision of manuscript. CH and FV: acquisition of data All authors contributed to the article and approved the submitted version.

## Funding

This article is supported by grants from the National Institutes of Health R01 HL149452 and HL156152-01A1 Catalyze.

## Conflict of interest

CH, FV, and YO have no commercial or financial relationships that could be construed as a potential conflict of interest. Z-JL and OV declared the following potential conflicts of interest with respect to the research, authorship, and/or publication of this article: the E-selectin gene modification technologies described in referenced articles by Z-JL and OV were developed in our research laboratory and patented/licensed by the University of Miami. This technology is currently under pre-clinical development by Ambulero Inc., a new start-up company out of the University of Miami that focuses on developing new vascular treatments for ischemic conditions. Co-authors, Z-JL and OV serve as consultants and scientific and medical advisory officers in Ambulero Inc.

## Publisher’s note

All claims expressed in this article are solely those of the authors and do not necessarily represent those of their affiliated organizations, or those of the publisher, the editors and the reviewers. Any product that may be evaluated in this article, or claim that may be made by its manufacturer, is not guaranteed or endorsed by the publisher.

## Abbreviations

AEs: Adverse Events
ABI: Ankle-Brachial Index
AD-MSCs: Adipose-Derived MSCs
AFS: Amputation-Free Survival
AR: Amputation Rate
ASM: Angiographic Score of MRA
Ang: Angiopoietin 1
BM-MNCs: Bone Marrow-Derived Mononuclear Cells
BM-MSCs: Bone Marrow-Derived MSCs
GCP-2: Chemokine Granulocyte Chemotactic Protein-2
CLTI: Chronic Limb-Threatening Ischemia
CXCR4: C-X-C Motif Chemokine Receptor 4
DR: Death Rate
DFU: Diabetic Foot Ulcer
DM: Diabetes Mellitus
DSA: Digital Subtraction Angiography
EPO: Erythropoietin
ESL: E-selectin Ligand
ETT: Exercise Treadmill Test
ECM: Extracellular Matrix
ERK 1/2: Extracellular Signal-Regulated Kinases 1/2
FAK: Focal Adhesion Kinase
HCELL: Hematopoietic Cell E−/L-selectin Ligand
HA: Hyaluronan
HIF-1α: Hypoxia-Inducible Transcription Factor 1α
MTD: Maximum Tolerated Dose
MSCs: Mesenchymal Stem Cells
NRS: Numeric Rating Scale
PWD: Pain Free walking Distance
PS: Pain scale
PAD: Peripheral Arterial Disease
PD-MSCs: Placental-Derived MSCs
PDGF: Platelet-Derived Growth Factor
PRP: Platelet-Rich Plasma
QoL: Quality of Life
SPP: Skin Perfusion Pressure
SDF1-α: Stromal-Derived Factor-1α
TcPO2: Transcutaneous Oxygen Pressure
TALEN: Transcription Activator-Like Effector Nucleases
TWD: Treadmill Walking Distance
TWD: Total Walking Distance
UCB-MSCs: Umbilical Cord Blood Mesenchymal Stem Cells
UH: Ulcer Healing
US: Ulcer Size
UTCS: University of Texas Diabetic Wound Classification Scale
VEGF: Vascular Endothelial Growth Factor
VAS: Visual analog score
